# Exposure to nonanoic acid alters small intestinal neuroendocrine tumor phenotype

**DOI:** 10.1186/s12885-023-10722-8

**Published:** 2023-03-23

**Authors:** Bilal Almobarak, Vishal Amlani, Linda Inge, Tobias Hofving, Andreas Muth, Ola Nilsson, Martin Johansson, Yvonne Arvidsson, Erik Elias

**Affiliations:** 1 Sahlgrenska Center for Cancer Research, Department of Laboratory Medicine, Institute of Biomedicine, Sahlgrenska Academy at University of Gothenburg, Box 425, Gothenburg, 405 30 Sweden; 2 Department of Vascular Surgery, Sahlgrenska University Hospital, Gothenburg, 413 45 Sweden; 3 Institute of Medicine, Department of Molecular and Clinical Medicine, Sahlgrenska Academy, University of Gothenburg, Gothenburg, 405 30 Sweden; 4 Department of Surgery, Institute of Clinical Sciences, Sahlgrenska Academy at University of Gothenburg, Gothenburg, 405 30 Sweden; 5 Section for Endocrine and Sarcoma surgery, Department of Surgery, Sahlgrenska University Hospital, Gothenburg, 413 45 Sweden; 6 Department of Pathology, Sahlgrenska University Hospital, Gothenburg, 413 45 Sweden; 7 Department of Pathology and Genetics, Institute of Biomedicine, Sahlgrenska Academy, University of Gothenburg, Gothenburg, 405 30 Sweden

**Keywords:** Neuroendocrine, SI-NET, SINET, SBNET, Neuroendocrine, Small intestine, Olfactory receptor, OR51E1

## Abstract

**Background:**

Small intestinal neuroendocrine tumors (SI-NET) are highly differentiated and genetically stable malignant tumors, yet they often present with advanced metastatic spread at the time of diagnosis. In contrast to many other types of malignant tumors, primary SI-NET are often asymptomatic and typically smaller in size compared to adjacent lymph node metastases. This study explores the hypothesis that stimulating the chemosensing olfactory receptor 51E1 (OR51E1) decreases SI-NET proliferation suggesting a mechanism that explains a difference in proliferative rate based on tumor location.

**Methods:**

Clinical data was used to address difference in tumor size depending on location. A SI-NET tissue microarray was used to evaluate expression of OR51E1 and olfactory marker protein (OMP). Primary cultured tumor cells from 5 patients were utilized to determine the effect of OR51E1 agonist nonanoic acid on metabolic activity. The SI-NET cell line GOT1 was used to determine effects of nonanoic acid on the transcriptome as well as long-term effects of nonanoic acid exposure with regards to cell proliferation, serotonin secretion, alterations of the cell-cycle and morphology.

**Results:**

Tumor size differed significantly based on location. OR51E1 and OMP were generally expressed in SI-NET. Primary SI-NET cells responded to nonanoic acid with a dose dependent altered metabolic activity and this was replicated in the GOT1 cell line but not in the MCF10A control cell line. Nonanoic acid treatment in GOT1 cells upregulated transcripts related to neuroendocrine differentiation and hormone secretion. Long-term nonanoic acid treatment of GOT1 cells decreased proliferation, induced senescence, and altered cell morphology.

**Conclusion:**

Our results raise the possibility that exposure of intraluminal metabolites could represent a mechanism determining aspects of the SI-NET tumor phenotype. However, we could not causally link the observed effects of nonanoic acid exposure to the OR51E1 receptor.

**Supplementary Information:**

The online version contains supplementary material available at 10.1186/s12885-023-10722-8.

## Background

Small intestinal neuroendocrine tumor (SI-NET) is the most common malignant tumor of the small intestine [1] and is often diagnosed at a late stage with advanced metastatic spread. This presents a challenge in the management of SI-NET patients as late-stage disease precludes curative treatment. As a possible contributing factor to late diagnosis, SI-NETs in the intestine are often small and asymptomatic and symptoms are more often related to metastatic spread. Curiously, local lymph node metastases are commonly observed to be larger in size than intestinal primary tumors despite developing at a later stage [2]. SI-NETs are well differentiated and genetically stable with a low mutational burden [3] [4] and there are no established driver mutations explaining SI-NET initiation and progression [5]. Consequently, there are no established genetic mechanisms explaining the observation that SI-NETs seem to proliferate less efficiently in the intestine compared to local mesenterial lymph-nodes.

SI-NET are believed to originate from enterochromaffin cells (EC cells) [6] located as single cells among enterocytes along the intestinal tract. They produce and secrete gut derived serotonin (5-HT). Enteric serotonin secretion has been functionally linked to diverse mechanisms such as bowel motility, immune defense, and enterocyte proliferation [7, 8]. Enteroendocrine cells, including EC cells, are believed to act as chemosensing cells by utilizing receptor mediated detection of specific components in luminal content such as odorants, nutrients or microbial metabolites and responding with hormone synthesis and secretion [9] [10]. Olfactory receptors have been detected in non-olfactory tissue and expression of olfactory marker protein (OMP) has been proposed as a marker for olfactory receptor activity [11]. The Olfactory Receptor 51E1 (OR51E1) is expressed in enteroendocrine cell [12, 13] and has recently been shown to function as a chemosensing receptor that regulates serotonin secretion from EC cells [9, 10].

OR51E1 expression seems to be retained in SI-NET [14] and is found in other forms of neuroendocrine tumors [15] as well as in prostate cancer [16] that can display a neuroendocrine phenotype. Prostate cancer cell line experiments utilizing a previously validated OR51E1 agonist nonanoic acid and the structurally related non-agonist 1-nonanol [17, 18], induced cellular senescence and thus decreased tumor proliferation [19].

Antiproliferative olfactory receptor activity induced by intraluminal content represents a novel concept that could explain the difference in tumor size based on tumor location. Utilizing patient data, cultivated primary SI-NET tumor cells and the GOT1 cell line we performed an explorative study based on the hypothesis that olfactory receptor mediated EC-cell chemosensing mechanisms decreases SI-NET tumor proliferation.

## Methods

### Patient material

*Tissue Microarray (TMA)* The TMA contained 846 tumor biopsies from 412 patients retrieved from patients who underwent surgery for SI-NET at Sahlgrenska University Hospital in the years 1986 to 2013.

Details of the construction of the tissue microarray have previously been described [20]. The diagnosis of all tumors was revalidated by staining all tumors for hematoxylin and eosin, synaptophysin and chromogranin A, and reviewed by board certified pathologist (O.N.).

*SI-NET biobank* Fresh-frozen tissue biopsies were collected at the time of surgery from the Endocrine unit of the Surgery department at Sahlgrenska University Hospital from 1986 to 2019. Resected tissue from SI-NET patients was evaluated in the operating theatre and material was collected from primary tumors, lymph node and liver metastases. The extent of tissue sampled depended on tumor spread and size of resected tumors. Consistently, the largest intestinal tumor and the most adjacent lymph-node metastasis were sampled if present. Starting in 2010, measurements in mm of all 3 dimensions were obtained and documented in a local register coupled to the biobank. From 2010 to the present, tissue has been sampled by a group of three lab technicians in a standardized manner. Tumor volume (mm^3) based on measurements was estimated as = ((width (mm) * height (mm) * depth (mm)) / 2.

*Cell lines and patient-derived tumor cells* The GOT1 cell line was established from a liver metastasis of a midgut neuroendocrine tumor [21] and was cultured in RPMI1640 supplemented with 10% fetal bovine serum (FBS), 5 µg/mL insulin and 5 µg/mL transferrin. The P-STS cell line was a gift from Professor R Pfragner. It was established from the primary tumor, described as a grade 3 NET located in the terminal ileum [22], and was cultured in M199: Ham’s F12 (1:1) supplemented with 10% FBS. The cell lines were regularly tested for *Mycoplasma* species (Eurofins GATC Biotech GmbH) The identity of the cell lines was validated by STR analysis [23]. MCF10A was purchased from ATCC (CRL-10,317) and was cultured as previously described in Debnath et al. [24]. Patient-derived tumor cells were established from SI-NET samples stored in the local biobank and prepared as previously described [25]. All patient-derived tumor cells were cultured RPMI1640 supplemented with 4% FBS, 5 µg/mL insulin and 5 µg/mL transferrin. All culture media contained 100 IU/mL penicillin and 100 µg/mL streptomycin.

### Drugs and experimental methods

*Drugs and reagents* Nonanoic acid, 1-nonanol and decanoic acid were purchased from Sigma-Aldrich and dissolved in DMSO. Octreotide Hospira, 100 mg/ml injection solution was purchased from Pfizer.

*Immunohistochemistry (IHC) and Scoring of TMA* IHC was performed on cell lines GOT1, P-STS and MCF10A, patient tumor tissue and TMAs. Paraffin embedded cell blocks from all cell lines were created using a Cellient automated cell block system (Hologic). Paraffin embedded tissue blocks from those patient tumors used for primary cell cultures were obtained from Sahlgrenska University Hospital. These blocks were prepared for routine clinical histopathology. TMAs were generated as described above. Sections (3–4 μm) from paraffin blocks were placed on glass slides and treated in Dako PT-Link using EnVision FLEX Target Retrieval Solution (high pH). The following primary antibodies were used: anti-chromogranin A (PHE5, Millipore and ab68271, Abcam), anti-synaptophysin (Sy38, Dako), anti- OR51E1 (LS-A1854, LSBio) and anti-OMP (B-6, Santa Cruz). IHC staining was performed in a Dako Autostainer Link using EnVision FLEX according to the manufacturer’s instructions (DakoCytomation). EnVision FLEX+ (LINKER) rabbit or mouse was used for all staining’s except anti-chromogranin A (PHE5) Positive and negative controls were included in each run. Relative expression of OR51E1 and OMP on tumors present on the TMA was evaluated by assigning a score of 0–3 to the relative staining intensity, with 0 being negative staining and 3 the highest staining present on the TMA. This evaluation was performed by two blinded independent observers with an initial 87.6% agreement in assessment of OR51E1 score and 90.3% agreements in assessment of OMP score. Samples with differing assessments were reviewed again by both observers and a final score was agreed upon. Only overall staining intensity was assessed and subcellular staining was not assessed.

*Metabolic activity* The cells were seeded onto black non-optical 96-well cell culture plates (Nunc, Thermo Fisher Scientific) and 24 h was allowed for cell attachment before start of the experiments. The cells were treated with a range of 0-3000 µM nonanoic acid or 1-nonanol. To measure the metabolic activity after 3 days treatment the assay plates were incubated with AlamarBlue (Thermo Fisher Scientific) for 6 h at 37 °C, and then analyzed by a fluorescence plate reader (Wallac 1420, PerkinElmer; ex. 560 nm and em. 640 nm). All experiments were performed in triplicate with 3 technical replicates.

*Proliferation* GOT1, P-STS and MCF10A cells were treated with nonanoic acid, 1-nonanol or DMSO vehicle for at least two doubling times. Cells cultivated without drug were included as a control. For GOT1 this represents three weeks, for P-STS 10 days and for MCF10A 5 days. CyQUANT® Cell Proliferation Assay Kit (Invitrogen) was used according to manufacturer’s instructions to quantify DNA content reflecting cell amount. All experiments were performed in triplicate with 3 technical replicates.

*Serotonin quantification* GOT1 cells were cultured with medium containing nonanoic acid (300 or 750 µM) or medium with vehicle (DMSO). After 24 h exposure the medium was collected and serotonin was quantified using ELISA according to the manufacturer’s instructions (ab133053, Abcam). Medium with vehicle was included as a control. Experiments were performed three times with 3 replicates.

*Cell cycle analysis* For analyses of cell cycle s-phases, GOT1 cells were cultured in the presence of 300 µM nonanoic acid or medium with vehicle (0.1% DMSO) for 3 weeks. One million cells per ml were lysed and stained for 30 min at 37 °C in modified Vindelöv’s solution (20 mM Tris, 100 mM NaCl, 1 µg/mL 7-AAD, 20 µg/mL RNase, and 0.1% NP40 adjusted to pH 8.0) followed by the analysis of DNA content using the FL3 channel with a BD Accuri C6 flow cytometer. Experiments were performed three times with 3 replicates.

*Western blot analysis* Western blot was performed on GOT1 cells cultured with 300 µM nonanoic acid for 1, 2 or 3 weeks. Whole-cell lysates were prepared by adding ice-cold RIPA lysis buffer (Thermo fischer) and Protease Inhibitor Cocktail Set III (cat. no.: 539,134; EMD Biosciences, La Jolla, CA, USA). A total of 20 µg cell lysate was run on 10% or 4–12% NuPAGE Bis–Tris polyacrylamide gels (Invitrogen) and transferred to PVDF membranes (Invitrogen). The membranes were probed using the antibody anti-synaptophysin (DAK-SYNAP, Dako). Membranes were stripped with ReBlot Plus Strong Antibody Stripping Solution (Millipore, Temecula, CA, USA) and re-probed with antibody against β-actin (cat. no. MAB 8226; Abcam) to estimate the amount of protein transferred. Blotted proteins were visualized using HRP-conjugated secondary antibodies and chemiluminescence detection (Super Signal West Dura Extended Duration Substrate; Thermo, Rockford, IL, USA). The chemiluminescence signals were detected with an image reader (LAS 4000; Fujifilm, Tokyo, Japan) and quantified using MultiGauge version 3.1 software (Fujifilm). 5 replicates for 3 experiments were included. Relative arbitrary densitometric units (ADU) was normalized according to loading control (β-actin) and calculated as = (synaptofysin ADU)/ (β-actin ADU). Relative change in synapthofysin expression was calculated as = (nonanoic acid treatment normalized ADU)/ (mean DMSO control normalized ADU). Controls are presented as = (individual normalized control ADU / mean normalized control ADU).

*RNA sequencing and RT-PCR* GOT1 cells were cultured for three days with 300 µM nonanoic acid or medium with vehicle (0.1% DMSO).Cells were harvested and RNA extracted using RNeasy mini kit (Qiagen). RNA sequencing was performed at the bioinformatics core facility, Sahlgrenska academy, University of Gothenburg. Alignment, identification and quantification of transcripts enabling pairwise comparisons were performed using the Partek flow software. RNA from three replicate experiments of GOT1 cells exposed to 300nM Nonanoic acid or vehicle (DMSO) for three days were reverse-transcribed using Thermoscientific RevertAid RT kit (Thermofisher). PCR reactions in triplicate were set up using predesigned KiCqStart SYBR® Green primers for the genes ACTB, GAPDH, MDK, PCSK1, SCL18A1, SCL18A2, TPH1, VGF (Sigma-Aldrich) and SYBR™ Green PCR Master Mix (Applied Biosystems). The PCR reactions were subjected to PCR cycling conditions recommended by Applied Biosystems in a 7500 Fast Real-Time PCR System (Applied Biosystems). Expression values were calculated as mean values relative to ACTB and GAPDH (2^–(Ct (target gene)) – (Ct (ACTB+GAPDH/2))^). Statistical significance between experimental conditions was calculated using paired Student’s t-test (GraphPad Prism 8) and a p value of < 0.05 was considered as significant.

### Statistics

#### The following statistical tests were used

Wilcoxon sign rank test, Spearman test, Man-Whitney U, 2way Anova with Sidaks multiple comparisons, Oneway Anova with Tukeys multiple comparisons. Students T-test. The GraphPad Prizm8 software was used for all statistical calculations.

## Results

### Primary SI-NETs are smaller than paired adjacent lymph node metastases

**Fig. 1:**
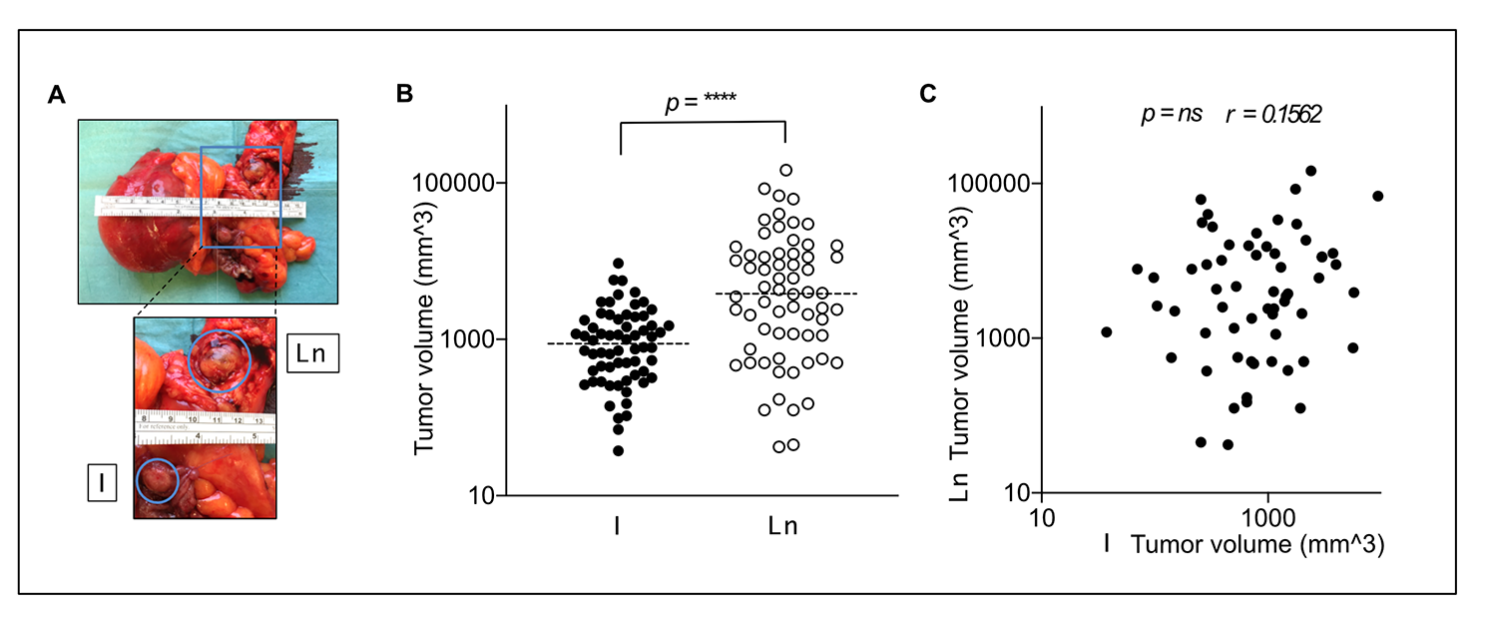
Tumor volume of intestinal SI-NET in comparison to matched adjacent lymph node metastases. **(A)** Image depicting SI-NET tumors, intestinal (I) and lymph node metastases (LN). **(B)** Volume of intestinal (I) and lymph node metastasis (LN) differ significantly, p < 0.0001, dotted line denotes mean. **(C)** Volume of paired intestinal (I) and lymph node metastasis (LN) do not correlate

We initially sought to evaluate the anecdotal clinical observation that primary SI-NETs are smaller in size compared to local lymph node metastases (Fig. 1A). A retrospective evaluation of all sampled SI-NETs stored at our local biobank (n = 342 patients) identified 64 patients that had (a) sampled the largest intestinal tumor (b) sampled the most adjacent lymph node metastasis and (c) had documented measurements of all three dimensions in both samples. Intestinal primary tumor volume was significantly smaller than adjacent lymph node metastasis, p < 0.0001, Wilcoxon sign rank test) Fig. 1B and tumor volume of paired intestinal tumors and adjacent lymph node metastases did not correlate (n = 64 pairs, r = 0.1562, p = 0.2177, Spearman test), Fig. 1C.

### OR51E1 and olfactory marker protein (OMP) are generally expressed in SI-NET

**Fig. 2:**
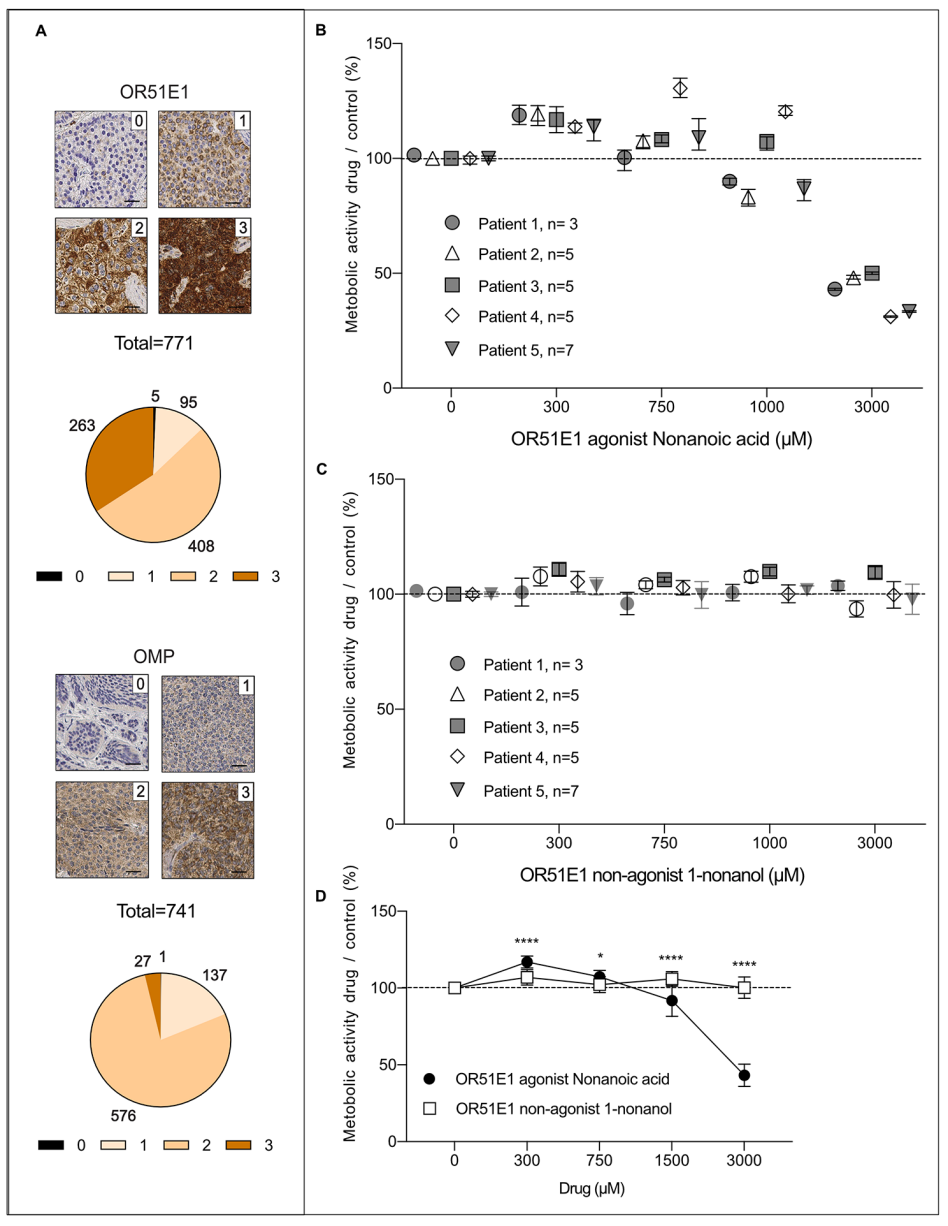
SINETs express high levels of OR51E1 and OMP proteins and nonanoic acid increases the metabolic activity in primary cultured SI-NET cells. **(A)** Representative examples and distribution of protein expression score of OR51E1 and OMP in SI-NETs evaluated by IHC in a TMA. **(B)** Effect of OR51E1 agonist nonanoic acid on metabolic activity in primary cultivated SI-NET cells from 5 patients (control = DMSO vehicle), n is the number of replicates and the graph depicts a symbol at the mean and bars depict range **(C)** Effect of non-agonist 1-nonanol on metabolic activity in primary cultivated SI-NET cells from 5 patients (control = DMSO vehicle), n is the number of replicates and the graph depicts a symbol at the mean and bars depict range. **(D)** Pooled results (all patients and replicates), symbol at mean and bars depict standard deviation, **** = p < 0,0001, *=p < 0,05

We then evaluated the IHC expression of olfactory receptor 51E1 (OR51E1) and olfactory marker protein (OMP), a marker for olfactory receptor activity [10], utilizing a large previously assembled tissue micro array (TMA). The staining pattern of OR51E1 was consistent with previous reports [13] with generally diffuse cytoplasmic staining and in some cases an accented perinuclear staining. The staining pattern of OMP was a diffuse cytoplasmic staining. Staining intensity was assessed by assigning a relative score of 0–3 to each sample. Regarding OMP scoring, 0.13% had score 0, 18.49% had score 1, 77.73% had score 2 and 3.64% had score 3. Regarding OR51E1 scoring, 0.65% had score 0, 12.32% had score 1, 52.92% had score 2 and 34.11% had score 3, Fig. 2A. OR51E1 and OMP scores were significantly higher in intestinal SI-NET compared with unpaired lymph node metastases (OR51E1 score I vs. Ln, p = 0.0014 and OMP score I vs. Ln p = 0.0001, Man-Whitney U, supplementary Fig. 1B).

### The OR51E1 agonist nonanoic acid induces a dose-dependent response in metabolic activity in primary cultured SI-NET cells

As SI-NET generally expressed OR51E1 and OMP we assessed if primary cultured SI-NET cells responded to the exposure of an OR51E1 agonist. Primary tumor cells from 5 SI-NET patients (supplementary Fig. 1A and supplementary Table 1) were treated for 72 h with 5 different concentrations of the OR51E1 agonist nonanoic acid, 1-nonanol (a structurally related non-agonist [18]) and medium with vehicle (DMSO). When normalized against the control, 300 µM of nonanoic acid significantly increased metabolic activity in all patients compared with 1-nonanol suggesting that low doses of nonanoic acid did not decrease viability but rather induced a specific energy consuming response (individual data Fig. 2B and C, aggregate data (Fig. 2D) with metabolic activity nonanoic acid/control vs. metabolic activity 1-nonanol/control, 2way Anova with Sidaks multiple comparisions, p < 0,0001 for drug concentrations of 300, 1500 and 3000 µM, p = 0,0301 for drug concentration of 750 µM, Fig. 2C). Higher concentrations (1500 and 3000 µM) of nonanoic acid decreased the metabolic activity interpreted as reduced viability due to non-specific toxicity.

### Nonanoic acid exposure in cell lines GOT1, P-STS and MCF10A

**Fig. 3:**
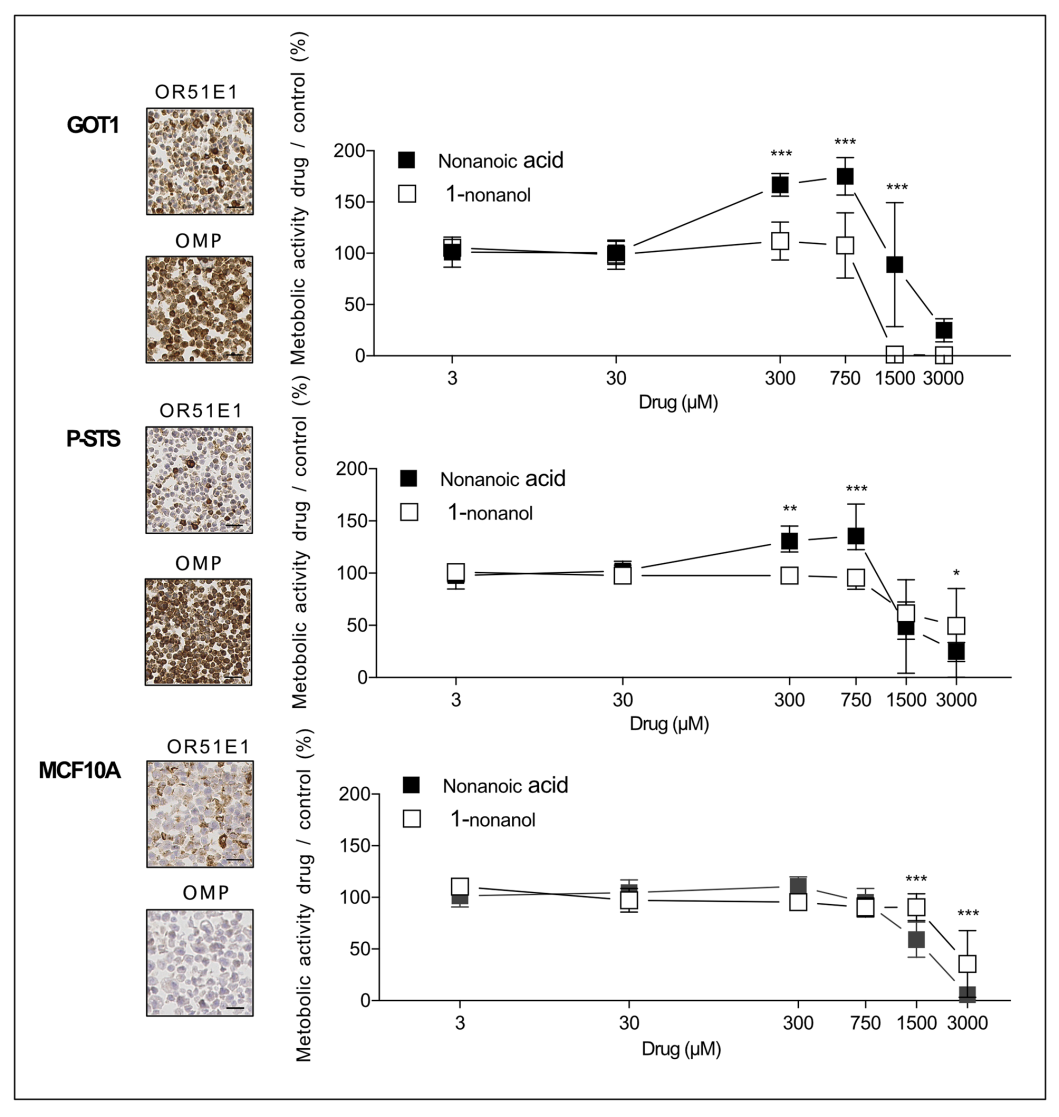
Nonanoic acid increase the metabolic activity in SINET cell lines. Expression of OR51E1 and OMP in SI-NET cell line GOT1, SI-NEC cell line P-STS and breast cancer cell line MCF10A/MCF evaluated by IHC on cellblocks. Scale bar is equal to 25 μm. Effect of OR51E1 agonist nonanoic acid compared to structurally related non-agonist 1-nonanol (control = DMSO vehicle) on metabolic activity/rate in cell lines GOT1, P-STS and MCF. Graph depicts a symbol at mean and bars depict standard deviation, *=p < 0,05, **=p < 0,01, ***=p < 0,001

As primary SI-NET cell cultures have limitations due to a mixture of cell types and cannot be maintained for longer time periods, we evaluated if the SI-NET cell line GOT1 was representative for further experiments. OR51E1 and OMP were strongly expressed in the SI-NET cell line GOT1 and in the SI-NEC (neuroendocrine carcinoma) cell line P-STS and weakly expressed in the breast cancer cell line MCF10A (Fig. 3). Both GOT1 and P-STS but not MCF10A responded to nonanoic acid with increased metabolic activity in a dose-dependent manner, (metabolic activity nonanoic acid/control vs. metabolic activity 1-nonanol/control, 2way Anova with Sidaks multiple comparisons, GOT1 p < 0,001 for drug concentrations of 300, 750 and 1500 µM, P-STS p < 0,001 for 750 µM, p < 0,01for 300 µM and p < 0,05 for 3000 µM, MCF10A p < 0,001 for 1500 and 3000 µM, Fig. 3).

The GOT1 cell line also responded to another proposed OR51E1 agonist [25], Decanoic acid, in a highly similar way (supplementary Fig. 2A).

### Short term effects of OR51E1 agonist exposure in GOT1 cells

**Fig. 4:**
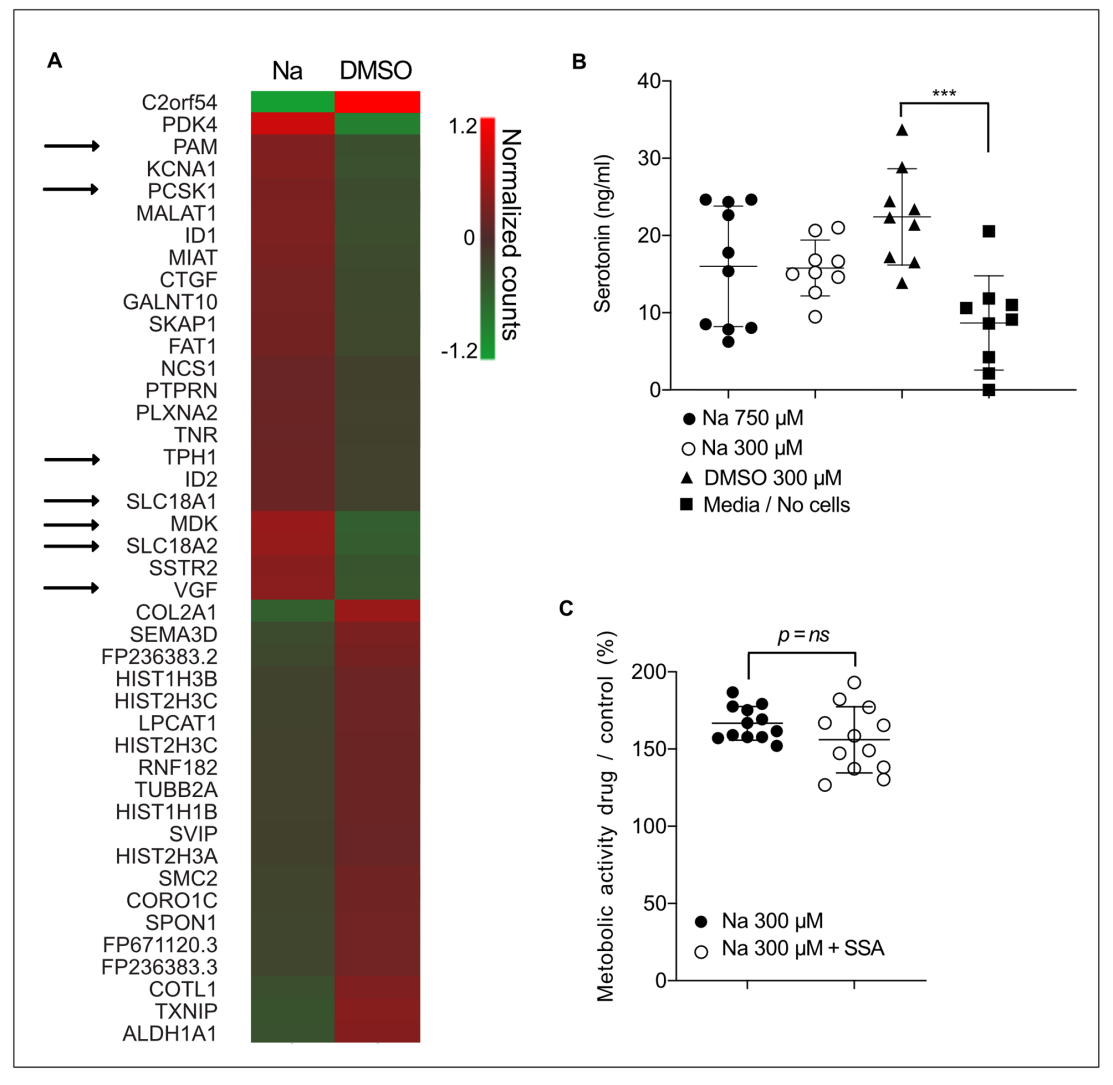
Effects of nonanoic acid in GOT1. **(A)** Transcripts that were significantly upregulated (p < 0.05) in response to nonanoic acid (Na) treatment in GOT1 cells. Arrows denote transcripts directly related to EC-cell hormone synthesis and secretion. **(B)** Secretion of serotonin measured by ELISA show serotonin secretion by GOT1 cells, but this was not affected by nonanoic acid exposure, symbols depict individual values, line depicts mean, and bars depict standard deviation, ***=p < 0,001. **(C)** Somatostatin-analog (SSA) did not affect the metabolic activity of GOT1 cells when treated with nonanoic acid (control = DMSO vehicle), symbols depict individual values, line depict mean, and bars depict standard deviation

As the OR51E1 agonist nonanoic acid induced an increased metabolic activity in both primary cultured SI-NET and GOT1 cells we aimed to determine if this alteration induced changes relating to proliferation and hormone secretion. Initially, GOT1 cells were cultivated with 300 µM nonanoic acid or medium with vehicle (DMSO) for 72 h and DNA amount was determined but there was no significant difference arguing against increased proliferation, (Oneway Anova with Tukeys multiple comparisons, relative DNA amount drug/control, nonanoic acid 300µ vs. 1-nonanol 300µ and nonanoic acid 750µ vs. 1-nonanol 750µ, p = ns, supplementary Fig. 2B). Furthermore, the observed effect of 300 µM nonanoic acid was consistent for 24, 48, 72 or 96 h, (2way Anova with Sidaks multiple comparisions, metabolic activity drug/ control, 24, 48, 72 or 96 h, p = ns, supplementary Fig. 2C). As SI-NET primarily secrete serotonin we assessed the effect of nonanoic acid, 1-nonanol or vehicle on serotonin secretion in GOT1 (nonanoic acid vs. 1-nonanol vs. vehicle, Oneway Anova with Tukeys multiple comparisons, p = ns and vehicle vs. media (no cells), p = 0,0002, Fig. 4B) but could not detect a significant difference. Somatostatin-analog (SSA) is used as a treatment in NET patients and reduces hormone secretion and tumor proliferation. Therefore, we evaluated if SSA affected the response to nonanoic acid but there was no significant difference (nonanoic acid vs. nonanoic acid + octreotide and 1-nonanol vs. 1-nonanol + octreotide, Oneway Anova with Tukeys multiple comparisons, p = ns, Fig. 4C). Additionally, GOT1 cells were cultivated with 300 µM nonanoic acid or medium with vehicle (DMSO) for 72 h, RNA was extracted, and RNA-sequencing was performed. Gene expression values were normalized and differentially expressed genes were identified by students t-test. Analysis of the transcriptome indicated a significant upregulation of several genes directly related to EC-cell hormone production and secretion [12] (indicated with arrows in Fig. 4A). Findings were verified using RT-PCR in 3 independent experiments (supplementary Fig. 3). We also performed a siRNA and shRNA mediated OR51E1 knockdown as previously described in other cell lines [9], [25]. However, despite achieving a significant decrease in OR51E1 transcripts, this did not translate into reduced protein levels of OR51E1 limiting the usefulness of this methodology to assess the correlation between protein levels and effects on metabolic activity and transcriptome alterations (*data not shown*). We also performed an experiment utilizing exposure of the proposed OR51E1 antagonist *Ethylhexanoic acid* (Eh) in a short term 72 h) experiment assessing if the combination of Eh with nonanoic acid blunted the increase in metabolic activity induced by nonanoic acid in GOT1 cells. We did not observe any statistically significant effect of adding Ethylhexanoic acid (supplementary Fig. 2D).

### Prolonged treatment with an OR51E1 agonist decreases tumor proliferation and promotes neuroendocrine differentiation

**Fig. 5:**
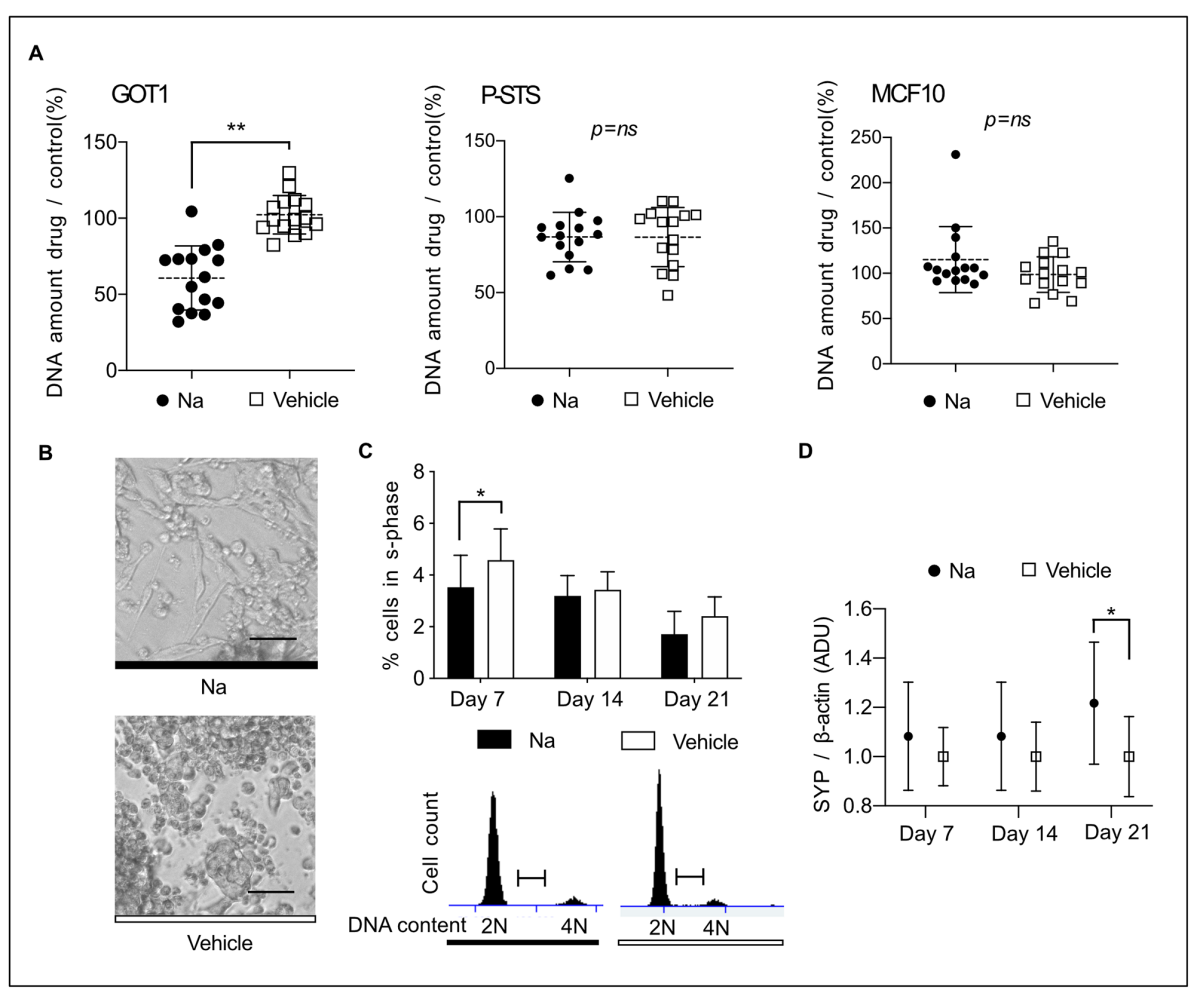
Prolonged nonanoic acid exposure could promote neuroendocrine differentiation. **(A)** Effect of nonanoic acid vs. vehicle (DMSO) treatment over 2 doubling times on cell proliferation (control = no drug), symbols depict individual measurements, line depicts mean, and bars depict standard deviation, **=p < 0,01. **(B)** Representative image of GOT1 cells exposed to nonanoic acid or vehicle (DMSO), for 3 weeks. Scale bar is equal to 100 μm. **(C)** Cell cycle analysis after 3 weeks nonanoic acid treatment versus DMSO in GOT1 cells, large bar depicts mean and error bar depict standard deviation, *=p < 0,05. **(D)** Effect of nonanoic acid treatment on synaptophysin expression in GOT1 cells (ADU = arbitrary densitometric units), symbol depicts mean, and bars represent standard deviation, *=p < 0,05

In accordance with our main hypothesis that continuous exposure to naturally occurring OR51E1 ligands in the intestine could reduce SI-NET proliferation we carried out a series of long-term experiments. To determine the effects of continuous exposure to nonanoic acid on proliferation in SI-NET we treated GOT1, P-STS and MCF-10 A cells with 300 µM nonanoic acid, 1-nonanol or medium with vehicle (DMSO) during approximately two doubling times. In GOT1 cells nonanoic acid decreased cell proliferation with approximately 50% compared with DMSO vehicle (nonanoic acid vs. vehicle, t-test, p = 0,0001, Fig. 5A). There was no effect of on proliferation in P-STS and MCF10 (nonanoic acid vs. vehicle, t-test, p = ns, Fig. 5A). Finally, 1-nonanol did not affect proliferation in any cell lines (*data not shown*). Also, in GOT1 cells, long-term exposure to 300 µM nonanoic acid decreased the s-phase consistent with cellular senescence (nonanoic acid vs. vehicle, 2way Anova with Sidaks multiple comparisons, p = 0,03 at 7 days, p = ns at 14 and 21 days, Fig. 5C) In addition prolonged exposure increased the expression of the neuroendocrine differentiation marker protein synaptophysin (nonanoic acid vs. vehicle (DMSO), 2way Anova with Sidaks multiple comparisons, p = 0,01 at 21 days, p = ns at 7 and 14 days, Fig. 5D) and altered the cell morphology towards an increasingly differentiated appearance (Fig. 5B).

## Discussion

We hypothesized that external factors decreasing SI-NET proliferation could explain the curious observation that SI-NET located in the intestine are commonly smaller than adjacent lymph node metastases. and that exposure to intraluminal metabolites targeting chemosensing receptors could represent an external factor that mainly affect tumors located in intestine. Although several of our observations are consistent with the hypothesis that external factors could affect SI-NET proliferation, our findings could not causally link these effects to the chemosensing OR51E1 receptor.

The concept of intraluminal metabolites directly affecting intestinal tumors has to our knowledge not been previously addressed in SI-NET but is an active research field in other intestinal epithelial malignancies such as colorectal cancer and gastric cancer. Diet and microbial composition have been indicated as contributing factors in both tumor initiation and proliferation [26, 27]. The EC cell and gut-derived serotonin has been implicated in a diverse set of physiological responses yet the exact mechanisms by which the EC cell interacts with its surroundings has been difficult to study as EC cells represents a minority of epithelial cells and are located as single cells dispersed along the small intestine. This has recently been circumvented by the use of intestinal organoids and several receptors expressed in the EC cell, including OR51E1 mouse homolog Olfr558, has been convincingly shown to detect various compounds and respond with voltage-gated Ca2 + channel-dependent serotonin release [9].

These observations coupled with previous reports that OR51E1 is expressed in SI-NET [14] and that targeting OR51E1 in prostate cancer cell lines induced senescence [19] prompted us to evaluate if SI-NET responded to exposure of an OR51E1 agonist. Primary cultured cells from 5 different SI-NET tumors responded with an increase in metabolic activity when exposed to 300 µM nonanoic acid indicating a dose dependent energy consuming cellular response consistent with increased receptor activity. Higher concentrations of nonanoic acid decreased metabolic activity suggesting reduced viability and we interpret this as a non-specific toxic effect possibly caused by a lowered pH. A concentration of 300 µM is roughly similar to reports of other cell lines responding to nonanoic acid [10, 19]. In order to explore the effects of nonanoic acid exposure in SI-NET we validated the GOT1 cell line that displayed a strikingly similar response with regards to an increased metabolic activity in response to nonanoic acid treatment. Interestingly, serotonin secretion in response to OR51E1 stimulation was however not increased possibly reflecting a dysregulation in SI-NET hormone secretion compared to the EC-cell. Even more interestingly, exposure to nonanoic acid affected the transcriptome by upregulating several transcripts directly related to hormone synthesis and secretion, some of which were unknown until a recent publication determined the EC-cell sectretome [13]. One of these were MDK which translates into the Midkine protein. Midkine has been studied in prostate cancer with neuroendocrine differentiation [28] and elevated blood levels of Midkine has been suggested as a biomarker in SI-NET [29]. We then studied the long-term effects of nonanoic acid exposure, theoretically mirroring a continuous exposure to OR51E1 agonists in intestinal tumors, and found that this significantly decreased proliferation and altered cell morphology.

Taken together, it is tempting to speculate that continuous exposure to OR51E1 agonists could drive intestinal tumors towards a more highly differentiated secretory phenotype with subsequent reduced proliferative activity. Although this is an appealing mechanism it must be noted that based on the present findings it remains an open question whether our observations are directly linked to OR51E1 receptor activity. As our siRNA and shRNA mediated OR51E1 knockdown did not result in decreased OR51E1 protein levels, we could not causally link the effects of nonanoic acid exposure with the presence of the OR51E1 receptor in our GOT1 cell line. There is a lack of representative cell lines for SI-NET and GOT1, which is the best characterized SI-NET cell line, is unfortunately notoriously difficult to utilize in experimental setups.

Also, the lack of an observed effect of a proposed OR51E1 antagonist could in fact argue against OR51E1 mediating the effects of nonanoic acid exposure that we have observed. Although previous reports utilizing other cell lines including other enteroendocrine cells [10] link nonanoic acid mediated OR51E1 activity to downstream cellular effects, there is a clear possibility that the effects of nonanoic are not dependent on activation of the OR51E1 receptor in SI-NET. Alternative explanations could include signaling via another receptor as exemplified by a recent publication reporting on antiproliferative effects in colorectal cancer of ketone bodies signaling via the Hcar2 receptor [30]. Alternatively, dietary composition and specifically high fat diet has been reported to induce a peroxisome proliferator-activated receptor delta signature in intestinal stem cells and drive tumor formation [31]. As malignant cells such as SI-NET can show phenotypical characteristics of stem cells, a possible mechanism of action for nonanoic acid could be interference with this type of mechanism.

The findings that nonanoic acid affects tumor phenotype and tumor proliferation is still consistent with the concept of intraluminal metabolites affecting tumor characteristics based on location. Nonanoic acid, also known as pelargonic acid, occurs naturally in many edible plants and it is believed that most people are regularly exposed to this substance.

It should also be noted that OR51E1 agonists have been detected in plasma [32], presumed to originate mainly from food digestion. However, it has also been suggested that OR51E1 agonists could be released by adipocytes[32] as a possible endogenous agonist. Further research is required to evaluate these observations in the context of SI-NET proliferation. Also, intestinal ketogenesis in enterocytes induced by high fat feeding has been linked to hormone secretion from enteroendocrine cells [33] indicating an additional mechanism by which intraluminal content and diet composition could affect enteroendocrine cells.

Finally, the strikingly altered morphology in GOT1 cells exposed to nonanoic acid is not mirrored in intestinal tumors when compared to lymph node metastases. This could perhaps be reconciled with the fact that most of our findings, although statistically significant, were relatively small in absolute terms. Given that primary tumors of SI-NET do proliferate, albeit at a slower speed than lymph node metastases, we suggest that a local inhibitory effect mediated by luminal content would provide a small relative effect that is amplified over time.

Although it is a novel concept in SI-NET tumor biology, several examples of non-mutational external factors affecting tumor proliferation exist in the context of other endocrine tumors, as an example, patients with disseminated differentiated thyroid cancer are commonly treated with supra-physiological doses of thyroxin resulting in suppression of thyroid stimulating hormone which has an inhibitory effect on tumor proliferation [34]. Also, first line treatment of disseminated gastro-entero-pancreatic NET includes somatostatin analogues that reduces hormone secretion but also, by an unknown mechanism, decreases tumor proliferation [35] thus establishing a link between hormone secretion and tumor proliferation in NET. Finally, the initiation of type 1 and type 2 gastric NET are causatively linked to increased levels of the gastrin hormone [36].

## Conclusion

The results presented in this paper suggests the possibility that intraluminal contents could affect the SI-NET phenotype. The mechanisms that mediate these effects at a cellular level should be further explored.

## Electronic supplementary material

Below is the link to the electronic supplementary material.

Supplementary Material 1

## Data Availability

As required by the obtained ethics permit, patient data that support the findings of this study are not publicly available to ensure that included patients cannot be identified. Further questions regarding patient data should be addressed to E.E. Data regarding cell culture experiments are available on request, E.E. The GOT1 RNA datasets generated and analyzed during the current study will be available in the GEO repository, accession GSE216067.
