# Supplementary material for: Exposure to nonanoic acid alters small intestinal neuroendocrine tumor phenotype
Source: BMC Cancer. 2023 Mar 23;23:267. doi: 10.1186/s12885-023-10722-8 (PMC10035137; doi:10.1186/s12885-023-10722-8)
Supplement: Supplementary file 1 — Supplementary Material 1 [file 12885_2023_10722_MOESM1_ESM.pdf]

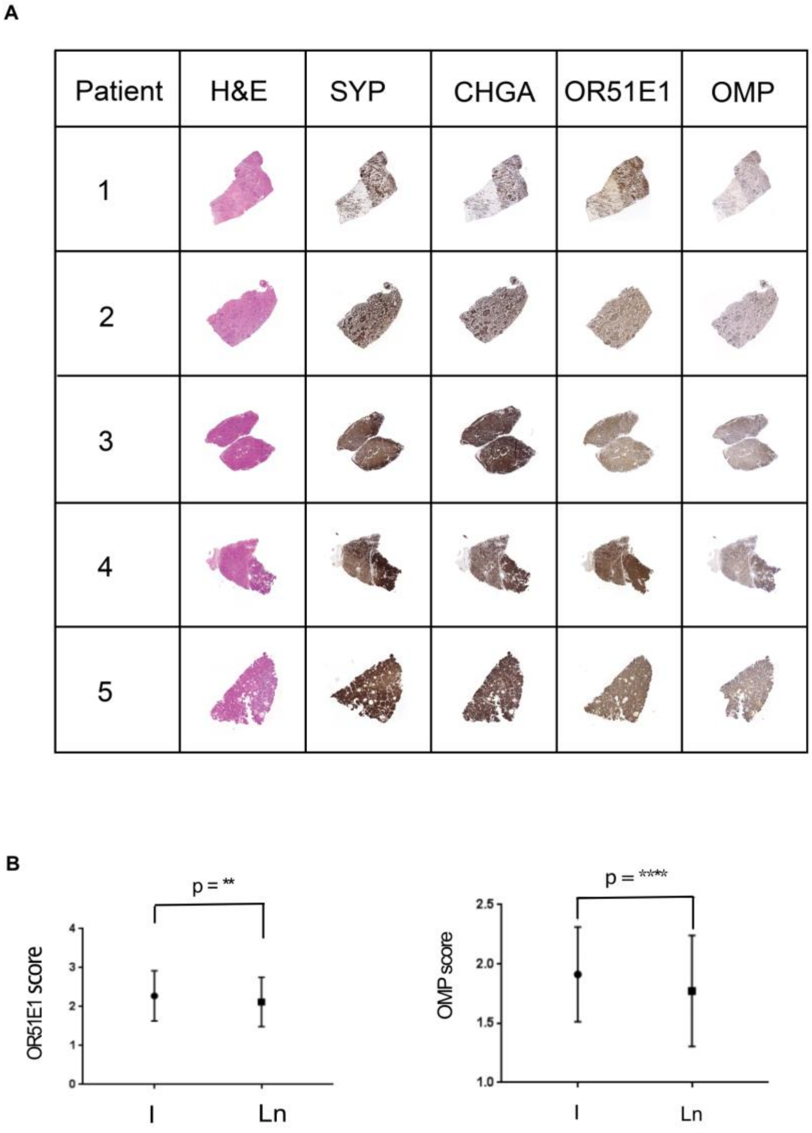

Supplementary Figure 1 A) IHC staining of SI-NET markers, OR51E1 and OMP. B) OR51E1 and OMP score in intestinal tumors (I) vs lymph nodes (Ln).

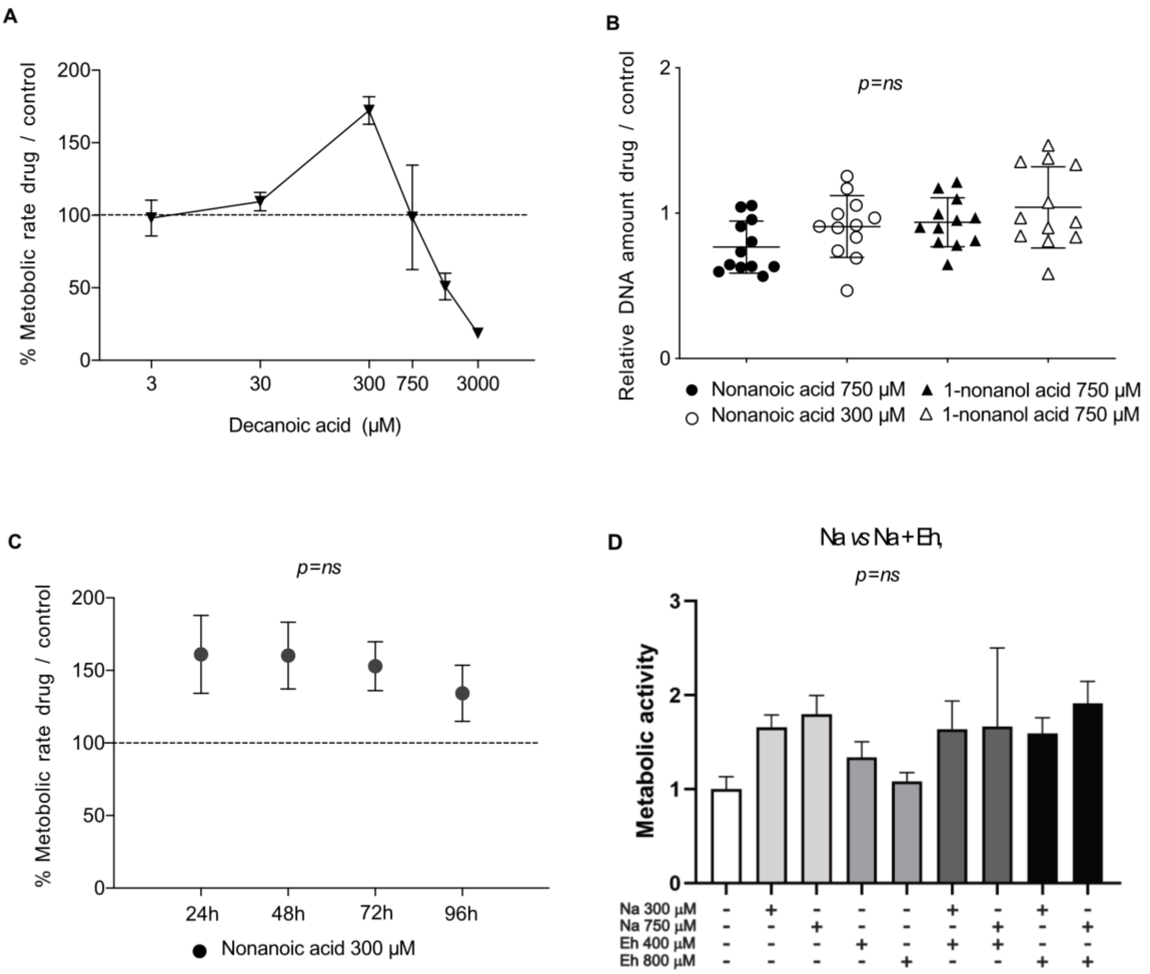

Supplementary Figure 2 A) Metabolic activity in response to decanoic acid in GOT1 cells (control = DMSO vehicle). B) Proliferation in response to short term (72h) nonanoic acid or 1-nonanol treatment (control = DMSO vehicle). C) Metabolic activity in response to 300 µM nonanoic acid in GOT1 cells over time (control = DMSO vehicle). D) Effect of Ethylhexanoic acid (Eh) in combination with Nonanoic acid (Na) on metabolic activity in GOT1 cells. Bars depict metabolic activity relative to control (DMSO vehicle), There was no significant effect of Na vs. Eh+Na, p=ns.

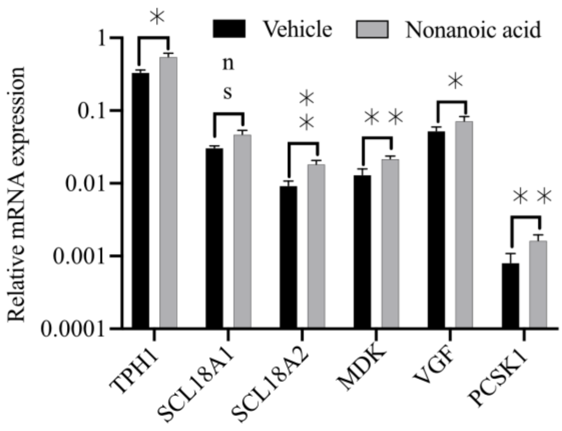

Supplementary figure 3. Bars represent the mean value (relative to GAPDH and ACTB) of 3 independent experiments and the error bars denote standard error of mean (SEM). Asterisks indicate statistically significant changes (paired student t test): * p < 0.05, ** p < 0.01

Supplementary Table 1

Details regarding primary cultured SI-NETs

Number	Tissue in experiment	Stage	Grade	KI67 (in small intestinal tumor)	mitosis/10 HPF
1	lymph node met	pT3(m)N1Mx	1	<1%	1
2	lymph node met	pT4N1MX	1	<1%	<1
3	lymph node met	pT4N2	1	<1%	<1
4	lymph node met	pT3N2Mx	2	4.28%	1
5	liver met	pT4N1M1	1	<1%	1
